# Power-Law Reliability Plotting for Microelectronics

**DOI:** 10.3390/mi16091055

**Published:** 2025-09-16

**Authors:** Joseph B. Bernstein

**Affiliations:** Department of Electrical and Electronic Engineering, Ariel University, Ariel 40700, Israel; josephbe@ariel.ac.il

**Keywords:** power-law, time domain, plotting, extrapolation, time to fail, TTF

## Abstract

The power-law time plotting for reliability prediction needs to be reexamined. Until now, most degradation plots in microelectronics reliability analysis assume that the data follow a power-law change in time. The plot is the change in a measured parameter versus the log of time, based on the principle that one can calculate exactly the initial indicator value, S_0_, and from that, extrapolate any change in that parameter, ΔS(t), as a power-law with time, t^1/m^. The normalized change, ΔS(t)/S_0_, relies heavily on a precise value for S_0_ such that the calculated power-law exponent, m, may be exaggerated such that extrapolated time-to-fail calculations will be optimistic, even by many orders of magnitude. Also, the extrapolated lifetime may be pessimistic, also by orders of magnitude in time. We show that by transforming the *x*-axis as the time to a power of 1/m, choosing m by setting the second order of a polynomial curve fit to zero, a more accurate prediction can be achieved with a realistic time to fail given the accelerated testing conditions. We also show how to determine what the correct power of time is using a linear fit to a second-order polynomial. The plotting principles presented here are independent of any physics, rather an empirical focus on how to plot the data according to a power-law in time assumption.

## 1. Introduction

Reliability data plotting is more of an art than a science. The goal of plotting raw data in a particular format is to match the science or theoretical physics underlying the phenomenon that is observed. It is important to understand that an assumed physics or degradation mechanism determines the plotting axes. The axes are determined based on the extrapolation of the expected degradation [1]. That is to say that the chosen axes prejudice the extrapolation based on an assumed theory. Often the theory will require that the extrapolation be according to a power law rather than a log-of-time *X*-axis. Nonetheless, the tendency is to use the log-of-time for the *X*-axis and transform the *Y*-axis data to be the log of a change in parameter versus the log of time. This way, the slope will indicate what the power-law is for the specific degradation mechanism being observed. For example, NBTI data is often plotted as the log of change in threshold voltage versus the log of time [2,3].

For many years, the International Reliability Physics Symposium, as well as other forums, has had scientists presenting degradation data and proving or disproving degradation mechanisms based on a developed theory fit to experimental data. Consistently, due to the basic assumption of thermodynamics and an Arrhenius theory of defect creation and propagation, the log-of-time axis is chosen, regardless of the theory that is applied. This is basically justified as it is consistent with Weibull plotting for failure probability, and it is consistent with most physics-based accelerated life-test principles. The assumption is that an expected time to fail will be accelerated on a logarithmic time axis with linearly increasing stress conditions, consistent with the Arrhenius law.

Joe McPherson, in his now classic book, “*Reliability Physics and Engineering, Time-To-Failure Modeling*,” has canonized today’s approach for plotting degradation based on a power-law assumption as a more generalized scheme compared to plotting on a semi-log graph [1]. Log of time is the *X*-axis; however, rather than a linear abscissa, which would work for the special case of a log-time dependence on stress, he suggested using a log of S(t), *Y*-axis. In principle, this assumption works out well for most failure data, as McPherson points out in his second chapter, since the data will generally not fit properly to a linear-log-of-time plot. Aside from this book, I could not find another microelectronics reference describing a different approach to extrapolate a time to fail from degradation data. This seems to be the definitive approach used ubiquitously in the literature, and there is no controversy or alternative.

The assumption for a degradation profile, according to McPherson, would follow the parameter, S(t), over time at a given stress level

(1) $$S(t)=S_{0}(1\pm ct^{1/m})$$

So to find the parameters S_0_, c, and m, we need to transform the parameters such that we can make a log–log plot of S(t) versus time. This is a very straightforward and common-sense way to find the divergence of the indication parameter, S(t), from some initially determined S_0_.

For the most part, this is the assumption of most reliability data plots in electronic devices, including silicon NBTI [2,3], HCI [4], SiC [5] devices, as well as GaN [6] transistor degradation. However, if we look at the required *Y*-axis, which is to be plotted on a log–log scale, we notice an interesting phenomenon that perhaps needs to be analyzed from a fresh perspective. According to McPherson, we plot X and Y such that

(2) $$X=\log\left(t\right)$$

and

(3) $$Y=\log\left[\frac{S\left(t\right)-S_{0}}{S_{0}}\right]=log\left(\frac{\Delta S}{S_{0}}\right).$$

At first glance, it looks perfectly fine, and it is the way our industry has been plotting their data for decades. By assuming that the line is straight, then you can calculate c and m directly from the curve. This allows extrapolation in time to an assumed failure criterion, when S(t) = S_F_, where S_F_ is the failure criterion. In general, when plotting ring oscillator frequency degradation, for example, NBTI data, this criterion may be 10% degradation (i.e., S_F_ = 1.1S_0_) [7], or it could be 20% degradation (1.2S_0_), as is typical for discrete power devices [8]. The calculation of the *Y*-axis value depends critically on an exact value for S_0._ The sensitivity of the calculations, including extrapolation of m and c, depends critically on an exact measure of S_0_ with nearly zero margin of error. Because S_0_ is both in the numerator and denominator of (3), the sensitivity is extremely high with respect to any variation on this parameter, S_0_.

## 2. Problem with Initial State Measurement

The assumption from Equation (1) is that the initial parameter remains mostly unchanged for the initial portion of the test and only slowly deviates over time. However, when plotting changes in this parameter on a log scale, where there is no ‘zero’ value on a log scale, the data becomes very awkward in the short times, where ΔS/S_0_~0. As a result, the plots can only begin when the data consistently shows parameter S(t) > S0 (or S(t) < S_0_ when measuring a decreasing parameter). Hence, we are limited to including data whose difference is above the noise margin of the measurements. As it turns out, the parameters are so sensitive to an exact measurement of S_0_ that any small noise margin will tremendously affect the calculations both for c and m, and the exponent will be generally much higher than the actual result, leading to orders of magnitude in error of time to fail with only fractions of a percent miscalculation in the exact S_0_.

**Figure 1:**
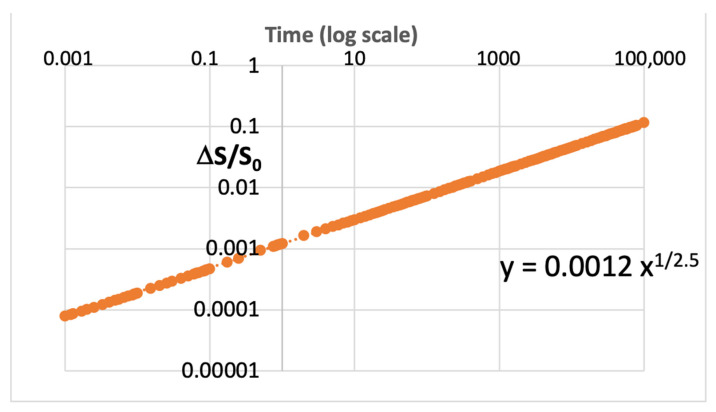
Illustrative ideal degradation parameter ΔS/S versus time on a log–log plot showing ideally how one can calculate c and m according to Equations (2) and (3) for X and Y.

This sensitivity can be best illustrated by example. Figure 1 shows synthesized data generated with a parameter, S(t), that has a power-law increase over time, consistent with the theory of Equation (1). The data mimics typical observed degradation characteristics seen in microelectronics. This dataset is plotted in Figure 1, and the relation for Y is shown with 1/m = 0.40, thus m = 2.5.

**Figure 2:**
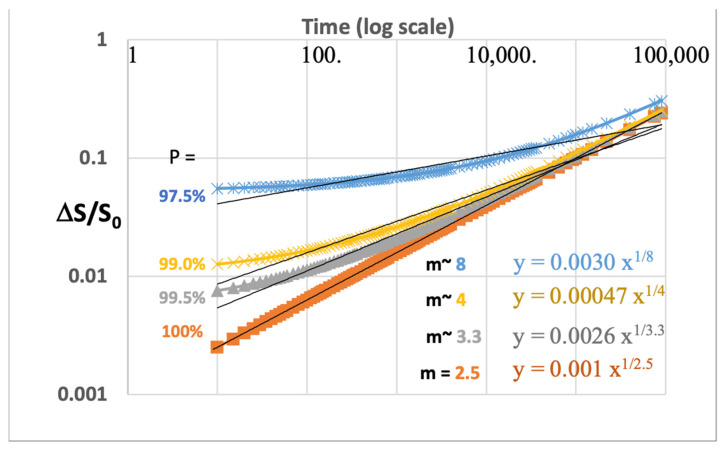
New values of c and m are calculated where S_0_ is multiplied by P from 97.5% to 99.5%. The ‘m’ values are written in the color of the corresponding line correlated with the resulting curve for each value of P shown.

Figure 2 shows the data, S(t), plotted with different values of S_0_, varied from its initial assumption by a small percent, from 97.5% of its actual value to as much as 99.5%. Figure 2 shows the same data as Figure 1, but where the value of S_0_ is multiplied by P. Again, this synthesized data illustrates the sensitivity of the initial value for S_0_ with very small variations in S_0_. These plots show the least squares calculations for m based on a linear fit from the log–log plots using Equation (3) for the Y value. We see that the values of m~8 in the root law for the time axis, where the error in S_0_ was only 2.5% (for P = 97.5%).

The plot of Figure 2 shows the dramatic effect that a very small deviation in the initial calculated parameter, S_0_, has on the resulting calculation for ‘m’ ranging from 2.5 to 8. On a log scale, the averaged value used in the plotting of ΔS/S_0_ will extrapolate to some assumption for S_0_ but never be exact since there is always noise associated with making that measurement. This alone can explain why often the power-law data is shown as ¼ or even as small as ^1^/_8_, depending on the uncertainty of that first point used for the calculation. Very often, there will be physics principles or theories as to exactly what exponent should be expected for this exponent. However, in our example, we show, without any theoretical motivation, that the power law of time can take on any value that would empirically be found from such a plot. Hence, the data plotting methodology is a critically important part of evaluating the time to fail or device lifetime under applied stress conditions.

## 3. Properly Plotting Power-Law Data

We need to know, with greater certainty, the correct power-law when extrapolating time to fail from degradation data. It is imperative to use an accurate plotting scheme to present the data such that there is no sensitivity error in finding the initial point, S_0_. There needs to be an accurate fit across most of the data so that a proper time-to-fail extrapolation can be achieved. Again, this is only when degradation theory dictates a power-law process causing the change over time. McPherson suggests that this would generally be the case. The proper transformation to make for Equation (1) would, thus, be to let

(4) $$X=t^{1/m}$$

and

(5) Y=S(t)

**Figure 3:**
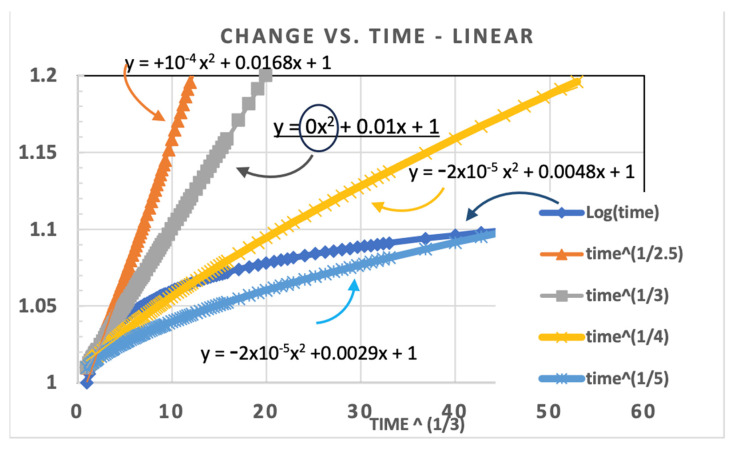
Simulated data of 0.01t^1/3^ plotted assuming m = 2.5 to 5 (labeled by color) with second order polynomial fit for each of the other assumptions showing a positive value for a too small m, and negative for too large m. The circle indicates that the x^2^ term is only zero for m = 3. The arrows indicate which polynomial was fit for each m value.

This way, when a least squares fit is made for Y versus X, we have a simple linear fit. Hence, with a properly fit ‘m’, a perfectly linear extrapolation can be made to any given failure criterion. However, there is one difficulty that must be resolved: that is to find the correct ‘m.’ This is accomplished by determining that the data forming the curve is actually straight, without any curvature. By inspection of Figure 2, we see that a curvature will result in an improper extrapolation. This makes sense since a curve represents a deviation from the assumed axes transformation, which can lead to a very large deviation if the curve were to continue. Therefore, the proper extrapolation must be perfectly straight along transformed axes. The curve shown in Figure 3 uses a different dataset than Figure 1 and Figure 2. Here, the power exponent was chosen as m = 3, not 2.5. This allows us to see the result of overestimating or underestimating the power-law using the same values for ‘m.’

For the trendline (in Excel), we used a second-order polynomial fit to compare different assumptions for which value of m correctly reflects the data trend. Figure 3 illustrates how we can compare different assumptions for ‘m’ and see the effect on the x^2^ term. Figure 3 shows that the simulated data is exactly Y = 1 + 0.01 t^1/3^ plotted on a linear Y versus X, where X = t^1/3^. We see in this plot that the x^2^ term is only zero when the plot is that of m = 3, but the x^2^ constant is negative for m > 3 (i.e., m = 4 or 5) and positive (+10^−4^) for m = 2.5 (i.e., m < 3). This way we know that the extrapolation of degradation versus time continues straight along the uncurved line with zero deviation. This figure also compares to the log-time function (in blue), which saturates and is never straight with a power-law transformation.

## 4. Application to Real Data

**Figure 4:**
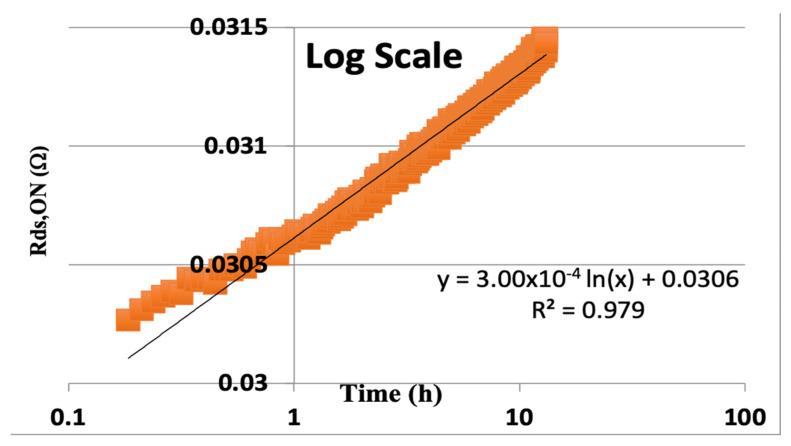
R_DS,ON_ versus time (hours) on a log scale.

We can now apply this methodology of extrapolating the time to failure based on real data that was collected from GaN power transistors from EPC corporation of El Segundo CA. The devices tested were EPC2016 and operated at 110 V (10% over specification) for 13 h. The ON state resistance (R_DS,ON_) was recorded versus time in hours and plotted on a log scale for time, as is traditionally performed. We see what looks like a very nice least squares fit to the data, as shown in Figure 4.

The data in Figure 4 shows the curve fit for this real data, which would extrapolate the time to fail (TTF) on a log scale to the time (x) when the change in resistance would be 20% of the initial resistance of 0.0306 Ohms. So, when 3.00 × 10^−4^ × ln(x) = 0.2 × 0.0306 = 6.12 × 10^−3^. Thus, the extrapolated time to 20% degradation is

(6) $$TTF=\exp\left(\frac{0.00612}{0.0003}\right)=e^{20.4}=7.2\times 10^{8}\;h$$

**Figure 5:**
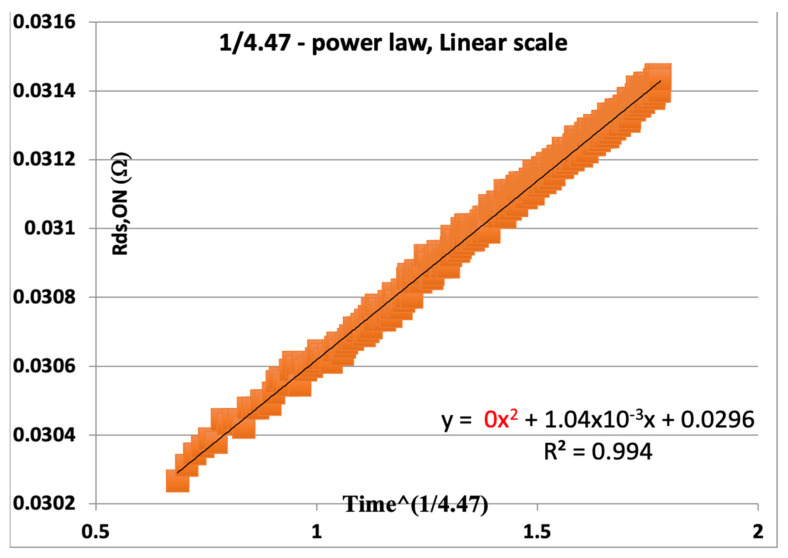
Same data as Figure 4 with a variable m to minimize the second-order term X^2^ so that there is zero curvature.

This result is equivalent to about 82,000 years! That is a very optimistic result if only it could be believed. However, we can replot this same data using our proposed calculation for the exponent of time, whereby the second derivative is fit to zero, as seen in Figure 5. Here, we minimized the x^2^ term by solving for zero, using the Excel solver function. We minimize the constant with m = 4.47. This polynomial fit took away the curvature of the plot, and we have a realistic extrapolation to time for 20% degradation.

We can now extrapolate a more realistic time to fail by finding X_F_ = TTF^1/4.47^ when R_DS,ON_ increases by 20% over the initial R_0_. From the least squares fit of the 2nd order polynomial using Excel, where we find m such that the x^2^ term is solved for zero (or as close to zero as possible), we have a proper value for the actual initial value for R_0_, which is 0.0296, and the proper slope on the new axis is 0.00104X (where X = t^1/4.47^). Therefore, we want to find X such that the increase in resistance is 20%, or 0.2 × 0.0296 = 0.00592, so

(7) $$X_{F}=\frac{0.00592}{0.00104}=5.69={TTF}^{\frac{1}{4.47}}$$

(8) $${{TTF=X}_{F}}^{4.47}=2377\;hours$$

This result, based on finding the appropriate time axis by eliminating the curvature of the data, results in two important insights into the expected time to fail:

1. *The predicted time to fail in hours is much more realistic for overvoltage testing (this is 10% above the rated voltage).*
2. *The extrapolation is based on a perfectly straight data extrapolation since we solved for the value, m, that brings the x^2^ term to zero, guaranteeing a linear fit.*

By choosing the time axis exponent properly and leaving the Y-axes linear, the dependence on initial value, S_0_, as in Equation (3), is eliminated. The result is that a more appropriate fit can be made based on the entirety of the data. In this one example, we see that the initially assumed value (R_0_, in our example) is 0.0306; however, when we use the entirety of the data, we find that the power-law is (1/m) such that m = 4.47. In this example, the real R_0_ based on this time exponent calculation is 0.0296, a value that is P = 96.7% of the originally assumed value. Thus, we can tell from the calculations of Figure 2 that our m-value determined by the classical approach, using Equations (2) and (3), would give a very incorrect result for the power-law and a huge overestimation, by far, for the actual extrapolated time to failure. As we saw before, this methodology will yield a more conservative extrapolation of the time to fail (TTF) and is likely to be correct.

## 5. Silicon NBTI Degradation Example

This same methodology can be applied to the most common application of reliability prediction in silicon devices. We have seen with real data taken at a high temperature, 140 °C, accelerated testing of 28 nm FPGA technology. These devices were configured to observe degradation of ring oscillators (RO). The principle here is that device degradation is observed by measuring the change in frequency over time with a given stress voltage. It is understood from the principles of negative bias temperature instability (NBTI) degradation that negative bias on the PMOS devices increases the charge in the gate and leads to a shift in the threshold voltage of all the PMOS being stressed [9]. The set of data presented here was from a 28 nm technology node FPGA from Xilinx. The initial frequency, F_0_, was taken as the average measured frequency for the first 40 min of operation. This value was then subtracted from the measured frequency over time, F(t).

The assumed degradation here is understood to be a decrease in the frequency over time, so we use Equation (1) to assume a power-law decrease in frequency over time. The difference in the frequency is then normalized by F_0_ as per Equation (3) to calculate Y. X, of course, is the time on a log scale. We see the data plotted as per the instructions of McPherson, and the data looks very linear on a log–log scale. Then we use the Excel trendline function to calculate the trend to extrapolate time to fail. We see what looks like an excellent and proper fit to a power-law trendline with y = 1.73 × 10^−4^ t^0.647^. We can then find that m = 1/0.647 = 1.5456.

In most cases, for example, in Naouss and Marc [2] and in many other examples of NBTI data plotting [3], the time to fail (TTF) is extrapolated by extending the power-law curve to 10% degradation, or when the trendline goes to 0.1 (10% degradation)

(9) $$TTF={\left(\frac{0.1}{1.73\times 10^{-4}}\right)}^{1.5456}=18,572\;h\sim 2\;years$$

**Figure 6:**
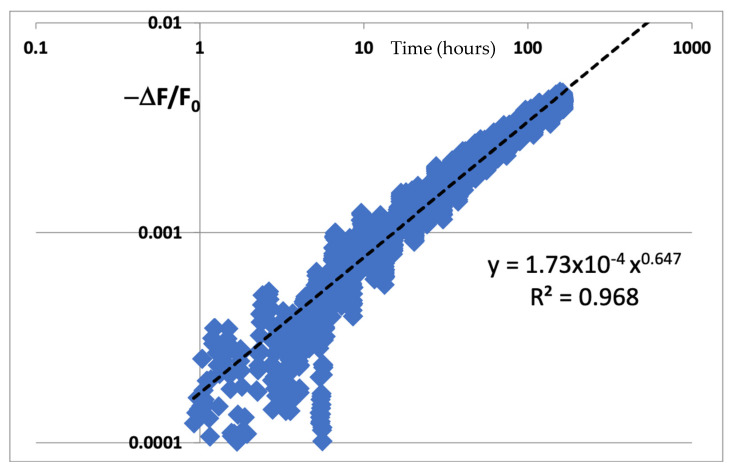
Plot of silicon NBTI data transformed as ΔF/F_0_ versus time on a log scale.

However, if we look carefully at the curve of Figure 6, it is possible to notice a slight bowing of the data with respect to the trendline. For the most part, like the GaN example shown above, this is easily overlooked since the R^2^ value is very good and there is the expected noise in the beginning of the data plot. Also, to fit the plot on a proper log–log scale, it was necessary to exclude some early data that were below zero due to the difference calculation for ΔF = F(t) − F_0_, since negative values are forbidden points on a log scale. Together, these two phenomena bring into question their entire calculation. Hence, we can compare our revised figure to a plot of the same data plotted linearly as a function of time to the power 1/m. From the data itself, we can minimize the curvature (not the R^2^ value) such that the line is perfectly straight. This is what we see next.

**Figure 7:**
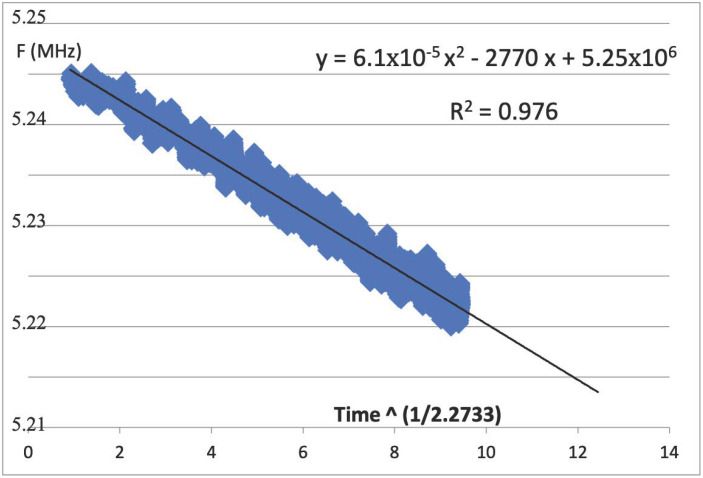
Plot of measured RO frequency in MHz versus time to the power 1/m.

In Figure 7, we see the trendline fit to a 2nd-order polynomial where the x^2^ term was solved to be as close to zero as is reasonable to achieve. For our data, 6.11 × 10^−5^ is for all practical purposes approximately zero. We see that the correlation coefficient, R^2^, is also quite good, but the power-law that was derived is m = 2.733. Thus, the proper way to plot this data is to have the actual frequency values for the *Y*-axis, as seen from Equation (5), while the *X*-axis should be the transformed time to the power 1/m, as we see from Equation (4); thus, Y = F(t), as the raw data from the measured frequencies directly from the FPGA.

We have here the raw data, F(t), plotted with the time axis. We can calculate the time to failure, TTF, as the time to 10% degradation from t = 0 (F_0_). That initial frequency here is seen as 5.25 MHz, while the slope is −2770 X. We now find the X value for which the trendline will reach Y = F_0_ × 0.9 (10% degradation in frequency). From here, we find

$$X_{F}=0.1\frac{5.25\times 10^{6}}{2.77\times 10^{3}}=189.5$$

(10) $$TTF={X_{F}}^{m}=189.5^{2.733}=1.68\times 10^{6}\;h=192\;years$$

In this example, we see that the correctly calculated TTF is nearly 100 times greater than the result from the classical calculation from Figure 6. This would suggest a severe undercalculation based on the traditional approach for extrapolating time-to-fail from degradation data. Thus, we see two examples of how properly determining the power time-law can give a drastically different result, either much greater or lower than what would have been found by transforming the *Y*-axis and leaving the *X*-axis simply as the log of time.

## 6. Discussion

We presented above two cases from real-world data that were normally used to extrapolate time-to-fail based on tracking the degradation and extrapolating based on a log-of-time assumption. The first example was R_DS,ON_ increase over time with high-temperature over-voltage stress. The voltage stress was 10% above nominal, which led to our calculation of about 2000 h, while the traditional McPherson approach gave thousands of years of extrapolated lifetime. The second case was 28 nm silicon technology testing NBTI degradation, where we saw that our simplified but more accurate approach gave a predicted lifetime that is 200 times greater than expected as reported from the traditional approach. Such enormous deviations will certainly leave great questions about the validity of any reliability data that extrapolates a time to fail based on degradation. The purpose of this paper is not to go through all the literature comparing how data is generally misinterpreted, but rather to raise awareness that it is not only the physics model that determines the predicted reliability but also the assumption of how the time axis is treated when extrapolating degradation to a time to fail.

## 7. Conclusions

Time extrapolation is about the most important goal of reliability testing and physics. It is well known that the assumed model for the physics determines the acceleration factor and extrapolated time to failure based on operating conditions. But more important than the physics alone is the proper selection of the time axis to determine times to fail from extrapolation of testing data. Since any reliability model must ultimately lead to a prediction for time to fail at a given set of operational conditions, the extrapolation itself due to degradation mechanisms must properly track this degradation. Therefore, we have shown that it is critical to choose the correct time-based model, and if a power-law time- based model is used for accelerated life testing, then it makes sense to transform the time axis such that the data is straight, whereby a proper linear extrapolation to TTF can be found. This is achieved by solving for the second-order term in a polynomial fit, selecting the 1/m power-law such that the x^2^ term goes to zero, guaranteeing a properly linear extrapolation. This will result in more consistent and realistic TTF predictions. Any subtle curvature of data on a log-of-time plot will lead to drastically incorrect results, being either optimistic or pessimistic by many orders of magnitude, putting into question nearly any extrapolation based on the classical approach.
